# A novel defined cuproptosis-related gene signature for predicting the prognosis of lung adenocarcinoma

**DOI:** 10.3389/fgene.2022.975185

**Published:** 2022-08-15

**Authors:** Huizhe Zhang, Yanchen Shi, Qing Yi, Cong Wang, Qingqing Xia, Yufeng Zhang, Weilong Jiang, Jia Qi

**Affiliations:** ^1^ Department of Respiratory Medicine, Yancheng Hospital of Traditional Chinese Medicine, Yancheng Hospital Affiliated to Nanjing University of Chinese Medicine, Yancheng, China; ^2^ Department of Pulmonary and Critical Care Medicine, Traditional Chinese and Western Medicine Clinical College of Nanjing University of Chinese Medicine, Nanjing, China; ^3^ Department of Pulmonary and Critical Care Medicine, Jiangyin Hospital of Traditional Chinese Medicine, Jiangyin Hospital Affiliated to Nanjing University of Chinese Medicine, Jiangyin, China; ^4^ Department of Pharmacy, Xin Hua Hospital Affiliated to Shanghai Jiao Tong University School of Medicine, Shanghai, China

**Keywords:** gene signature, cuproptosis, prognosis, lung adenocarcinoma, cilium

## Abstract

Lung adenocarcinoma (LUAD) has become the most prevalent histologic subset of primary lung cancer, and effective innovative prognostic models are needed to enhance the feasibility of targeted therapies for the disease. Programmed cell death (PCD) performs an integral function in the origin and treatment of cancer. Some PCD-related effective signatures for predicting prognosis in LUAD patients could provide potential therapeutic options in LUAD. A copper-dependent cell death referred to as cuproptosis is distinct from known PCD. However, whether cuproptosis is associated with LUAD patients' prognoses and the potential roles of cuproptosis-related genes involved is still unknown. For the prediction of LUAD prognosis, we developed a unique cuproptosis-associated gene signature. In The Cancer Genome Atlas (TCGA) cohort, the score derived from the risk signature on the basis of six cuproptosis-related genes was found to independently serve as a risk factor for anticipating lung cancer-related death. The differentially expressed genes between the high- and low-risk groups were linked to the cilium-related function. LUAD patients’ prognoses may now be predicted by a unique gene signature identified in this work. This discovery also provides a substantial foundation for future research into the links between cuproptosis-associated genes and cilium-related function in LUAD patients.

## Introduction

Lung adenocarcinoma (LUAD) is currently the most prevalent histologic subset of primary lung cancer, contributing to over 40% of all cases, and its relative prevalence is growing (Barta et al., 2019). The absence of adequate screening strategies and the challenges of performing an early diagnosis have resulted in significantly high recurrence and death rates for LUAD, with an overall five-year survival probability of lower than 15% owing to local and distant metastases (Ali et al., 2013). Despite great efforts having been made to explore the therapeutic effect of LUAD, the clinical outcomes of LUAD remain poor in patients (Zhang et al., 2015). Because of the limits of current LUAD therapies, novel treatment targets are required in order to improve the clinical result of LUAD. As a result, robust innovative prognostic models are needed in order to enhance the feasibility of targeted therapy for LUAD.

Programmed cell death (PCD) is critical for the appropriate development and maintenance of tissue homeostasis, as well as for the removal of damaged, diseased, or defunct cells in multicellular organisms. In the pathophysiology of different illnesses, aberrations in PCD signaling cascades, including ferroptosis, apoptosis, pyroptosis, necroptosis, and cell death linked to autophagy, may be detected (Galluzzi et al., 2018; Moujalled et al., 2021). Nowadays, several studies have established tumor prognostic models associated with PCD (Cai et al., 2021; Fu et al., 2021; Shao et al., 2021; Zhao et al., 2021). PCD performs an integral function in the origin and treatment of cancer (Shao et al., 2019; Strasser and Vaux, 2020). A few studies revealed crosstalk between distinct PCD mechanisms and antitumor immunity (Tang et al., 2020). Immunogenic cell death is a kind of tumor cell death that may be induced by some chemotherapy medicines, oncolytic viruses, physicochemical treatments, and radiotherapy (Ahmed and Tait, 2020). The ability of cancer cells to undergo death when subjected to anti-cancer therapy is mediated by modulated cell death systems, which could either suppress or enhance the immunogenic capacity of cancer cells (Garg and Agostinis, 2017).

The latest research shows that copper mediates cell death by targeting lipoylated tricarboxylic acid (TCA) cycle proteins, and because of this, lipoylated proteins aggregate, and corresponding iron-sulfur cluster proteins are lost, resulting in proteotoxic stress and eventually cell death. The authors demonstrate that copper-dependent modulated cell death in human cells is different from recognized cell death processes and is reliant on mitochondrial respiration (Tsvetkov et al., 2022). Therefore, this copper-dependent cell death was referred to as cuproptosis. There are very few in-depth studies on cuproptosis. There is a strong correlation between the abundance of ferredoxin 1 (FDX1) and the level of lipoylated proteins in a variety of human tumor cells, and cell lines with significant levels of lipoylated proteins are susceptible to cuproptosis, suggesting that copper ionophore intervention ought to be targeted toward malignancies with this metabolic landscape. Consequently, subsequent clinical studies of copper ionophores ought to be conducted utilizing a biomarker-driven strategy (Tsvetkov et al., 2022). According to the above studies on cuproptosis, we obtained cuproptosis-related genes including FDX1, dihydrolipoamide dehydrogenase (DLD), lipoic acid synthetase (LIAS), lipoyltransferase 1 (LIPT1), ATPase copper transporting beta (ATP7B), glycine cleavage system protein H (GCSH), ATPase copper transporting alpha (ATP7A), dihydrolipoamide S-acetyltransferase (DLAT), pyruvate dehydrogenase E1 subunit beta (PDHB), pyruvate dehydrogenase E1 subunit alpha 1 (PDHA1), dihydrolipoamide S-succinyltransferase (DLST), solute carrier family 31 members 1 (SLC31A1), and dihydrolipoamide branched chain transacylase E2 (DBT), which provide the preliminary basis for our next exploration and research.

Recently, a number of studies have established ferroptosis-, pyroptosis-, necroptosis- and autophagy-related effective signatures for predicting prognosis in LUAD patients, which could provide potential therapeutic options in LUAD (Chen et al., 2020; Lin et al., 2021; Yao et al., 2021; Lu et al., 2022). Given these existing findings, we hypothesized that cuproptosis is linked to LUAD patients’ prognoses and that cuproptosis-associated genes may be involved in the disease process. As a consequence, we conducted a comprehensive investigation to determine the expression patterns of cuproptosis-associated genes in normal lung and LUAD samples, ascertain the prognostic significance of these genes, and conducted the Gene Ontology (GO) and immune infiltration enrichment analyses.

## Materials and methods

### Datasets

We acquired the RNA sequencing (RNA-seq) data combined with the corresponding clinical features (phenotype and survival data) from the Genomic Data Commons (GDC) The Cancer Genome Atlas (TCGA) LUAD cohort, all of which were retrieved from the University of California, Santa Cruz (UCSC) Xena (http://xena.ucsc.edu)(Goldman et al., 2020). UCSC Xena is an analysis and visualization platform with excellent performance for both large public databases including TCGA (Chin et al., 2011) and the GDC (Grossman et al., 2016), and private datasets.

The RNA-seq data normalized to fragment per kilobase million (FPKM) values include the gene expression data of 59 normal and 526 tumor tissues samples (https://gdc-hub.s3.us-east-1.amazonaws.com/download/TCGA-LUAD.htseq_fpkm.tsv.gz; version 07-20-2019), the phenotype data include clinicopathologic features of 877 LUAD patients (https://gdc-hub.s3.us-east-1.amazonaws.com/download/TCGA-LUAD.GDC_phenotype.tsv.gz; version 08-07-2019) and survival data include survival time information of 738 LUAD patients (https://gdc-hub.s3.us-east-1.amazonaws.com/download/TCGA-LUAD.survival.tsv; version 07-20-2019) (Supplementary File S1).

### Identification of the cuproptosis-related gene expression levels and interactions

We extracted the 13 cuproptosis-related genes from the recent article (Tsvetkov et al., 2022). The downloaded expression data in the TCGA dataset were presented as FPKM values. The “limma” package (Ritchie et al., 2015) was used to identify differentially expressed genes (DEGs) related to cuproptosis between 59 normal and 526 tumor tissues, and a *p*-value < 0.05 was defined statistically significant difference. The “pheatmap” package was used to present the RNA levels of these 13 cuproptosis-associated genes. The following are the criteria for the genes linked to cuproptosis with differential expression: *p < 0.05, **p < 0.01, and ***p < 0.001. We employed these genes to create a protein-protein interaction (PPI) network with the Search Tool for the Retrieval of Interacting Genes/Proteins (STRING), version 11.5 (https://string-db.org/), which is a repository of PPIs that have been identified and anticipated. There are indirect (functional) and direct (physical) linkages resulting from computerized prediction, information transmission between organisms, and interplay obtained from other repositories (Szklarczyk et al., 2021). The minimum required interaction value for the PPI analysis was established at 0.15 (the lowest confidence level) due to the limited number of genes, which allowed for more interactions to be discovered. Visualization of the correlation network of these genes was accomplished utilizing the “igraph” package, which is a set of network analysis tools that have a focus on portability, efficiency, and simplicity of use.

### Tumor classification premised on the cuproptosis-related genes clusters

With the help of the R package “ConsensusClusterPlus” (Wilkerson and Hayes, 2010), a consensus clustering analysis of all 526 LUAD tumor tissues in the TCGA dataset was performed to examine the links between the expression of the 13 cuproptosis-related gene and LUAD subtypes. Once the clustering variable (k) was increased from 2 to 10, a suitable value of k could be found and the 526 LUAD tumor tissues were classified into suitable clusters on basis of the 13 genes, with the highest intragroup correlations and the low intergroup correlations. After collating the phenotype data and survival data and removing the missing values, the data on clustered gene expression and the clinical parameters encompassing the tumor, node, and metastasis (TNM) classification, age, gender, and survival status of 486 LUAD patients were presented via the “pheatmap” package, which allowed for visualization of the differences between the clinical parameters and the classified clusters. A comparison was made for the overall survival (OS) time among the divided clusters *via* the Kaplan–Meier (KM) survival analysis using the “survival” package.

### Development of a cuproptosis-associated gene prognostic model

In total, 526 LUAD specimens were paired with the 500 matching patients whose survival data was complete. To examine the predictive significance of the cuproptosis-associated genes, we conducted a Cox regression analysis on the data from the TCGA cohort to determine the relationships between each gene and survival status. We established *p* < 0.3 as the cut-off value to avoid missing important genes and identified genes associated with survival for subsequent evaluation. The least absolute shrinkage and selection operator (LASSO) Cox regression model was then employed to filter out the potential genes and to create the prognostic model, which was done with the help of the R package “glmnet” (Tibshirani, 1997; Engebretsen and Bohlin, 2019). The final decision was made to keep the genes along with their coefficients being retained, and the penalty parameter (λ) was chosen based on the bare minimum requirements. Using a linear combination for each prognostic survival-associated gene’s standardized expression level and its associated multivariate Cox regression coefficient (β), the risk score in the derivation, as well as the validation sets, were determined. The following was the equation for calculating the risk score: Risk Score = ∑(β_i_ × gene_i_ EXP) (EXP: normalized expression value). After classifying patients into low- and high-risk subgroups premised on their median risk scores, the OS duration was evaluated between the two subgroups utilizing the KM survival analysis method. Using the gene signature as a starting point, principal component analysis (PCA) was done with the help of the function “prcomp” in the R package “stats”. The R packages “survival” and “timeROC” were employed to execute analyses of patients’ distribution premised on the risk score, each patient’s survival status, KM survival curves, and receiver operating characteristic (ROC) curves.

### Assessment of the risk score’s independent prognosis

Patients with LUAD in the TCGA cohort were categorized into two subgroups premised on their median risk scores. The clinical features including TNM classification, age, gender, and survival status of LUAD patients in high- and low-risk subgroups were analyzed in conjunction with the risk score derived from our regression model. Univariate and multivariate Cox regression models were utilized in the investigation. After collating the phenotype data and survival data and removing the missing values, the survival-related gene expression data and the clinical parameters encompassing TNM classification, age, gender, and survival status of LUAD patients in low- and high-risk groupings were presented *via* the “pheatmap” package and the differences in the clinical parameters across the two groups were evaluated.

### Functional enrichment and immune infiltration analyses of the differentially expressed genes between the low- and high-risk subgroups

Patients with LUAD in the TCGA dataset were categorized into two subgroups premised on their median risk scores. Determining the DEGs that distinguished the patients at low and high risk was done using the criteria of |log2FoldChange (FC) | > 0.5 and adjusted *p* < 0.05. The “org.Hs.eg.db” program was utilized to obtain the entrezIDs of DEGs. By incorporating the “clusterProfiler” and the “GOplot” packages, we were able to conduct analyses of GO functional enrichment on the basis of these DEGs and entrezIDs (Yu et al., 2012; Walter et al., 2015; Wu et al., 2021). GO functional analysis comprises three classifications: biological process (BP), cellular component (CC) and molecular function (MF). Single-sample gene set enrichment analysis (ssGSEA) was performed with the help of the “GSVA” and “GSEABase” packages, which were employed to compute the infiltration scores of immune cells and to assess the functioning of immune-associated pathways (Hanzelmann et al., 2013).

### Statistical analysis

The gene expression patterns in the normal lung and LUAD samples were compared utilizing a single-factor analysis of variance, whereas the categorical data were evaluated utilizing the Pearson chi-square test. We used the KM survival analysis approach in conjunction with a two-sided log-rank test to assess the OS of patients across different subgroups. We employed univariate and multivariate Cox regression models to examine the risk model’s independent prognostic significance. Through the use of Mann–Whitney test, we evaluated the infiltrating levels of immune cells and the activation of the immune pathway between the two subgroups. RGUI 4.0.3 was employed to execute all analyses of statistical data.

## Results

### The cuproptosis-related gene expression levels and interactions

**FIGURE 1:**
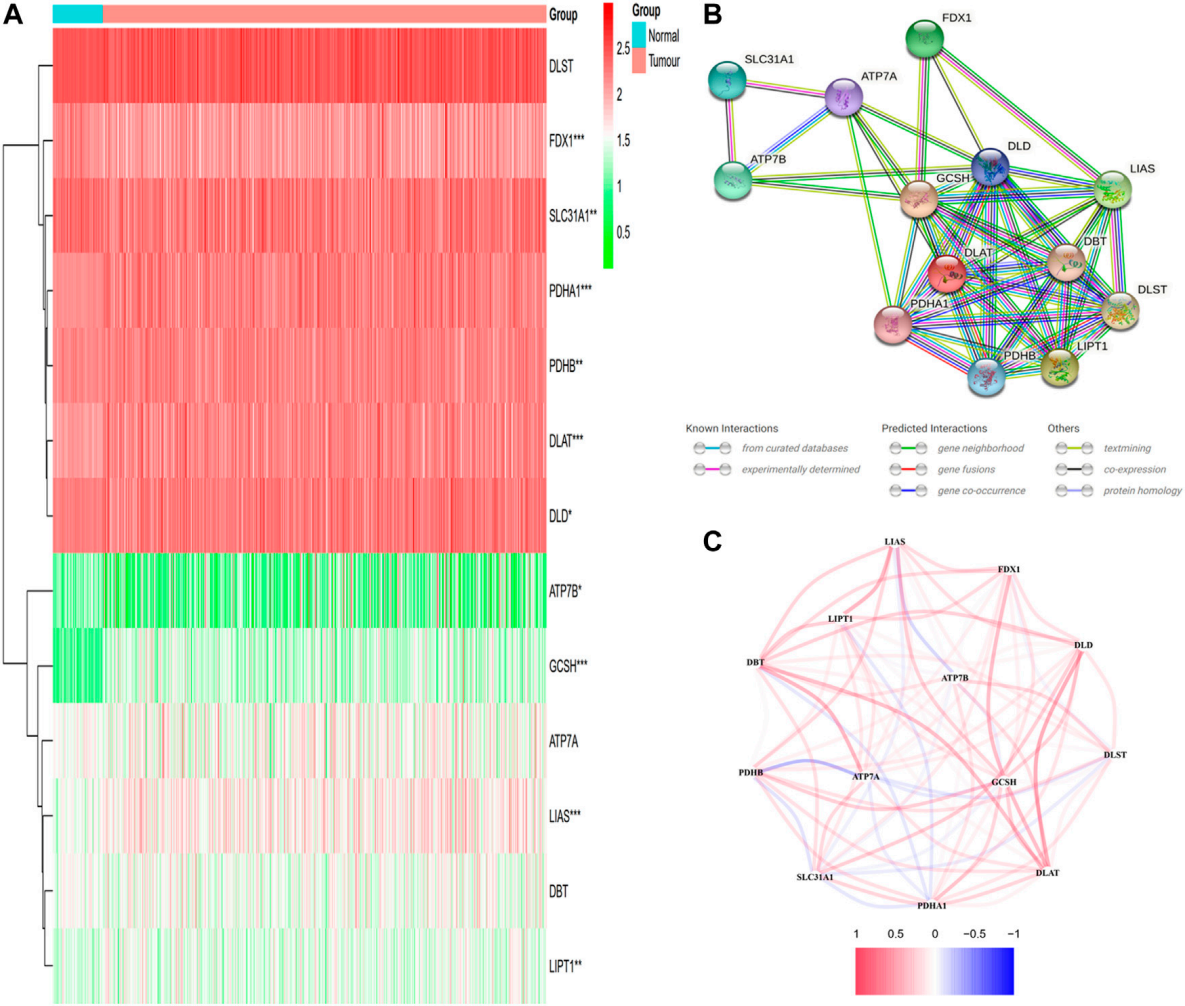
The expression and interactions of 13 cuproptosis-related genes. **(A)** The expression of cuproptosis-related genes in normal and tumor samples is shown in a heatmap (with green and red signifying low and high expression levels, respectively). Comparison between the tumor samples and the normal samples *p < 0.05; **p < 0.01; ***p < 0.001. **(B)** PPI network illustrating the connections of genes involved in cuproptosis (interaction score = 0.15). **(C)** The network of genes associated with cuproptosis (red and blue lines depict positive and negative correlations, respectively). The strength of relevance is reflected in the intensity of the colors.

The 13 cuproptosis-related gene expression levels were determined after comparing 59 normal and 526 tumor tissues in GDC TCGA LUAD cohort retrieved from UCSC Xena. Among them, 7 genes (DLAT, DLD, GCSH, LIAS, LIPT1, PDHA1, and PDHB) were upmodulated, whereas 3 genes (ATP7B, FDX1, and SLC31A1) were downmodulated in the tumor group in contrast with the normal group (*p* < 0.05). The levels of RNA for these 13 cuproptosis-related genes are displayed as a heatmap, where green and red denote low and high expression levels, respectively (Figure 1A). We undertook a PPI study on these cuproptosis-associated genes in order to learn more about their interactions with one another. Once the minimum required interaction score of the PPI analysis was adjusted to 0.15, the top five interaction proteins/genes were DLD, GCSH, DLAT, PDHA1, and LIAS, which could be considered as hub genes. Figure 1B illustrates the findings of PPI analysis. Figure 1C depicts the correlation network encompassing all genes associated with cuproptosis, where red and blue denote positive and negative correlations, correspondingly.

### Characterization of tumors depending on the genes involved in cuproptosis

**FIGURE 2:**
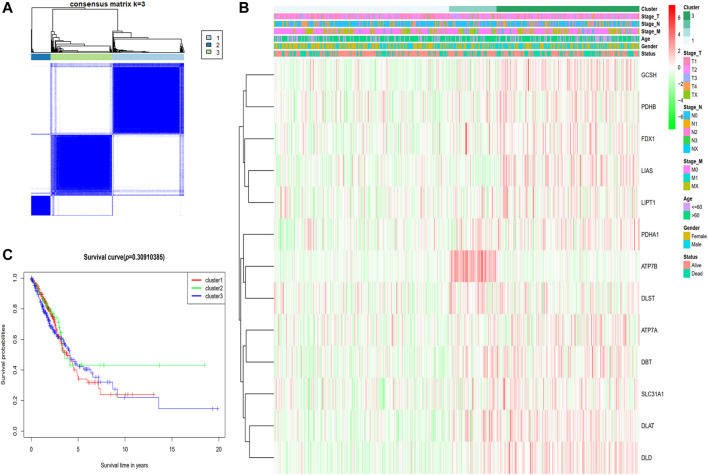
Tumor classification premised on the cuproptosis-associated genes. **(A)** Five hundred and twenty-six LUAD tumor samples were classified into three clusters utilizing the consensus clustering matrix (k = 3). **(B)** The heatmap depicting the clinical and pathological parameters of the three cuproptosis-related gene clusters. **(C)** Comparing OS time via KM survival analysis for the three clusters.

The expression of the 13 cuproptosis-associated genes was compared to the expression of LUAD subtypes utilizing a consensus clustering analysis of all 526 LUAD tumor specimens from the TCGA cohort in order to investigate the relationship between the two. After adjusting the clustering value (k) from 2 to 10, we discovered that at *k* = 3, the intragroup relationships were the strongest, illustrating that the 526 LUAD tumor tissues may be efficiently classified into three clusters on the basis of the 13 genes (Figure 2A). The 526 LUAD tumor tissues were corresponding to 486 LUAD patients with complete clinical features including TNM classification (stage N: N0, N1, N2, N3 or NX; stage M: M0, M1, or MX; stage T: T1, T2, T3, T4 or TX), age (≤60 or >60 years), gender (male or female) and survival status (dead or alive). An interactive heatmap is used to display the gene expression patterns and clinical parameters of 486 LUAD patients. The heatmap reveals that there are only minor differences in clinical parameters across the three clusters (Figure 2B). The KM survival analysis was utilized to measure the OS duration of the 500 relevant patients who had full survival time information among the three clusters, but differences were not obvious (*p* = 0.3091) (Figure 2C).

### Development of a prognostic gene model in the TCGA dataset

**FIGURE 3:**
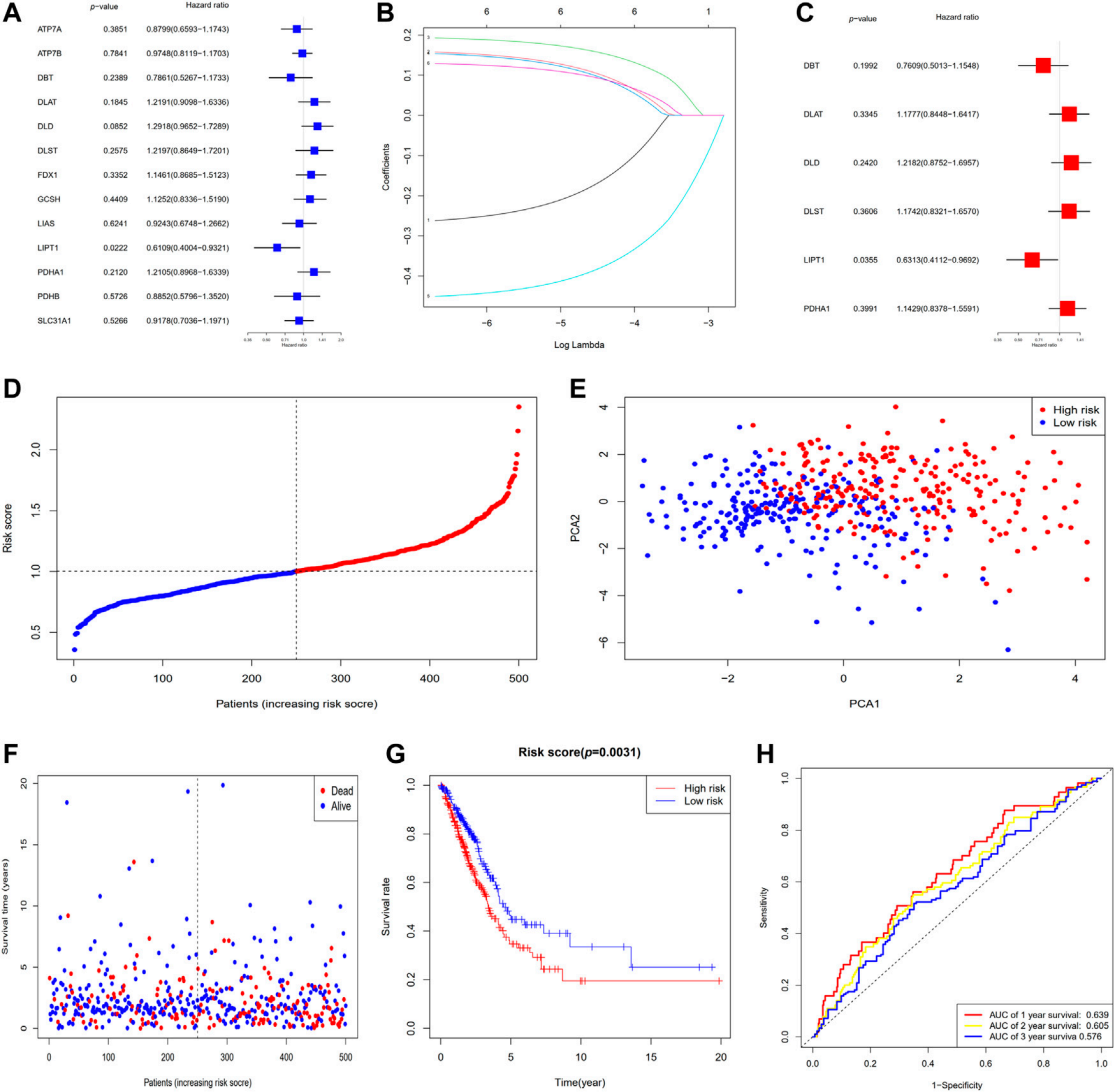
The establishment of a risk signature for the TCGA dataset. **(A)** OS analysis utilizing univariate Cox regression for each of the 6 cuproptosis-associated genes with *p* < 0.3. **(B)** LASSO regression analysis of the six genes associated with OS. **(C)** Analysis of OS utilizing multivariate Cox regression for the six genes associated with OS. **(D)** Patients are classified according to their risk scores. **(E)** PCA plot for patients with LUAD depending on their risk scores. **(F)** Each patient’s survival probability (low- and high-risk groups are displayed on the left and right sides of the dotted line, respectively). **(G)** KM survival study of patients classified as having high or low risks. **(H)** ROC curves proved the risk score's predictive effectiveness. AUC, area under the ROC curve.

In total, 526 LUAD specimens were paired with the 500 matching patients whose information on survival time was complete. The univariate Cox regression analysis was utilized for the initial filtering of the genes associated with survival. Subsequent investigations were conducted on the 6 genes (DBT, DLAT, DLD, DLST, LIPT1, and PDHA1) that satisfied the criterion of *p* < 0.3. Out of these 6 genes, 2 genes (DBT and LIPT1) were shown to have a protective function with hazard ratios (HRs) < 1, whereas the remaining 4 genes (DLAT, DLD, DLST, and PDHA1) were linked to a greater risk as demonstrated by HRs > 1. LIPT1 independently served as an influencing factor (*p* < 0.05) (Figure 3A). The Cox regression analysis conducted using the LASSO method was utilized to build a 6-gene signature that corresponded to the optimal λ value (Figure 3B). In the next step, we undertook a multivariate Cox regression analysis of the six genes (Figure 3C). The following is the equation for computing the risk score: risk score = (−0.2733 × DBT EXP) + (0.1636 × DLAT EXP) + (0.1974 × DLD EXP) + (0.1606 × DLST EXP) + (−0.4600 × LIPT1 EXP) + (0.1336 × PDHA1 EXP). Five hundred patients were separated into two groups depending on the median score generated by the risk score equation: low- and high-risk subgroups (Figure 3D). When the PCA was performed, it was determined that patients with different risks could be effectively categorized into two clusters (Figure 3E). A higher number of fatalities and a considerably shorter survival duration were observed among patients within the high-risk subgroup (displayed on the right-hand side of the dashed line) relative to those at low risk (Figure 3F). A notable difference (*p* = 0.0031) in OS duration was discovered between the high- and low-risk groups *via* KM survival analysis (Figure 3G). The prognostic model’s specificity and sensitivity were assessed utilizing time-dependent ROC curves. The findings indicated that the area under the ROC curve (AUC) for OS was 0.639, 0.605, and 0.576 for 1-, 2-, and 3-year periods, respectively (Figure 3H).

### The risk model’s independent prognostic significance

**FIGURE 4:**
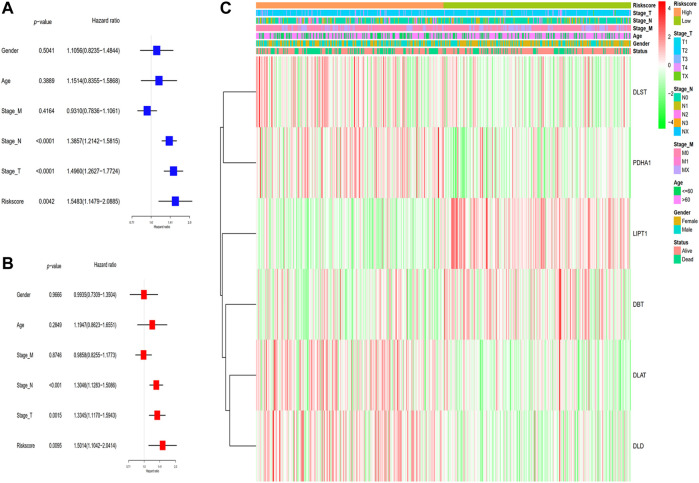
Analyses of the risk score utilizing univariate and multivariate Cox regression. **(A)** The TCGA dataset was subjected to a univariate analysis. **(B)** The TCGA cohort was subjected to a multivariate analysis. **(C)** Heatmap depicting the associations between clinical and pathological parameters and risk groups.

In the TCGA cohort, LUAD patients were classified into two subgroups based on their median risk score. The 482 corresponding patients had complete clinical features including survival status, TNM classification, age, and gender of LUAD patients in low- and high-risk subgroups were examined in combination with the risk score in the regression model. We utilized univariate and multivariate Cox regression analyses to ascertain the possibility of the risk score produced from the gene signature model independently serving as a prognostic factor. The analytical findings from the univariate Cox regression model illustrated that the risk score (HR = 1.5483, 95% CI: 1.973–5.467) independently functions as a predictive factor for unfavorable survival in the TCGA cohort (Figure 4A). Furthermore, after adjusting for possible confounders in the multivariate analysis, the risk score (HR = 1.5014, 95% CI: 1.1042−2.0414), stage T (HR = 1.3345, 95% CI: 1.1170−1.5943) and stage N (HR = 1.3046, 95% CI: 1.1283−1.5086) were found to be prognostic indicators for LUAD patients in TCGA cohort (Figure 4B). As an additional output of our analysis, we established a heatmap of clinical parameters for the TCGA dataset (Figure 4C), and the findings illustrated that the survival duration, stage T, and stage N were diversely distributed between the low- and high-risk subgroups.

### Gene ontology functional analysis premised on the risk model

**FIGURE 5:**
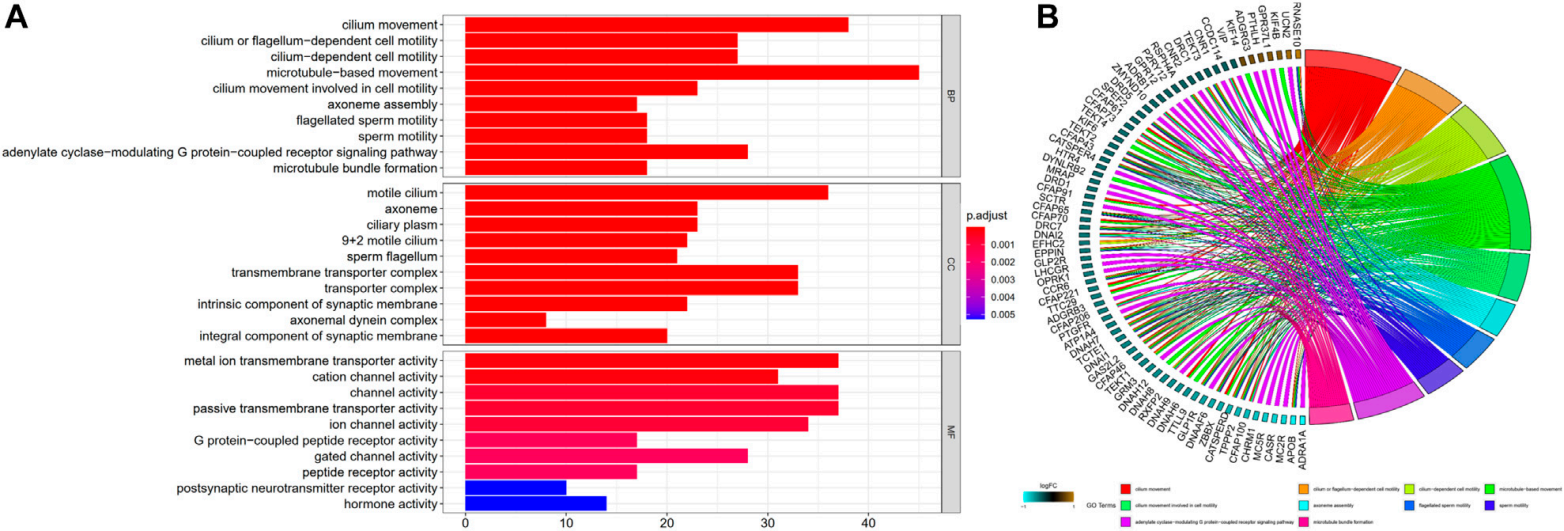
GO functional enrichments. **(A)** The top 10 GO functional enrichments included BP, CC, and MF. The smaller the adjusted *p*, the more significant the enrichment. **(B)** GO BP chord plot. GO terms represent the top 10 GO BP functional enrichments, and gene names with the connection represent their enriched genes. BP, biological process; CC, cellular component; MF, molecular function.

To additionally examine the difference in the functions of genes between the groups classified by the risk model, we employed the R function “limma” to obtain the DEGs premised on the cutoff values of adjusted *p* < 0.05 and |log2FC| > 0.5. Then, 1292 DEGs were detected between high- and low-risk subgroups in the TCGA dataset. 233 of them were upmodulated in the high-risk group, whereas the remaining 1,059 were downmodulated (Supplementary Table S1). Following that, a GO functional enrichment analysis was carried out using these DEGs. The function enrichment analysis of the GO BP illustrated a considerable enrichment of DEGs in cilium movement, cilium or flagellum-dependent cell motility, cilium-dependent cell motility, microtubule-based movement, cilium movement involved in cell motility and other processes. Analysis of the GO CC illustrated a substantial enrichment of DEGs in the motile cilium, axoneme, ciliary plasm, 9 + 2 motile cilium, sperm flagellum, and other components. The analysis of GO MF demonstrated a considerable enrichment of DEGs in metal ion transmembrane transporter activity, cation channel activity, channel activity, passive transmembrane transporter activity, ion channel activity, and other functions (Supplementary Table S2). The topmost 10 GO functional enrichments ordered by adjusted *p* are depicted in Figure 5A. The top 10 GO BP functional enrichments with their enriched genes are shown as GO chord plots (Figure 5B).

### Assessment of the immune activity across the groups

**FIGURE 6:**
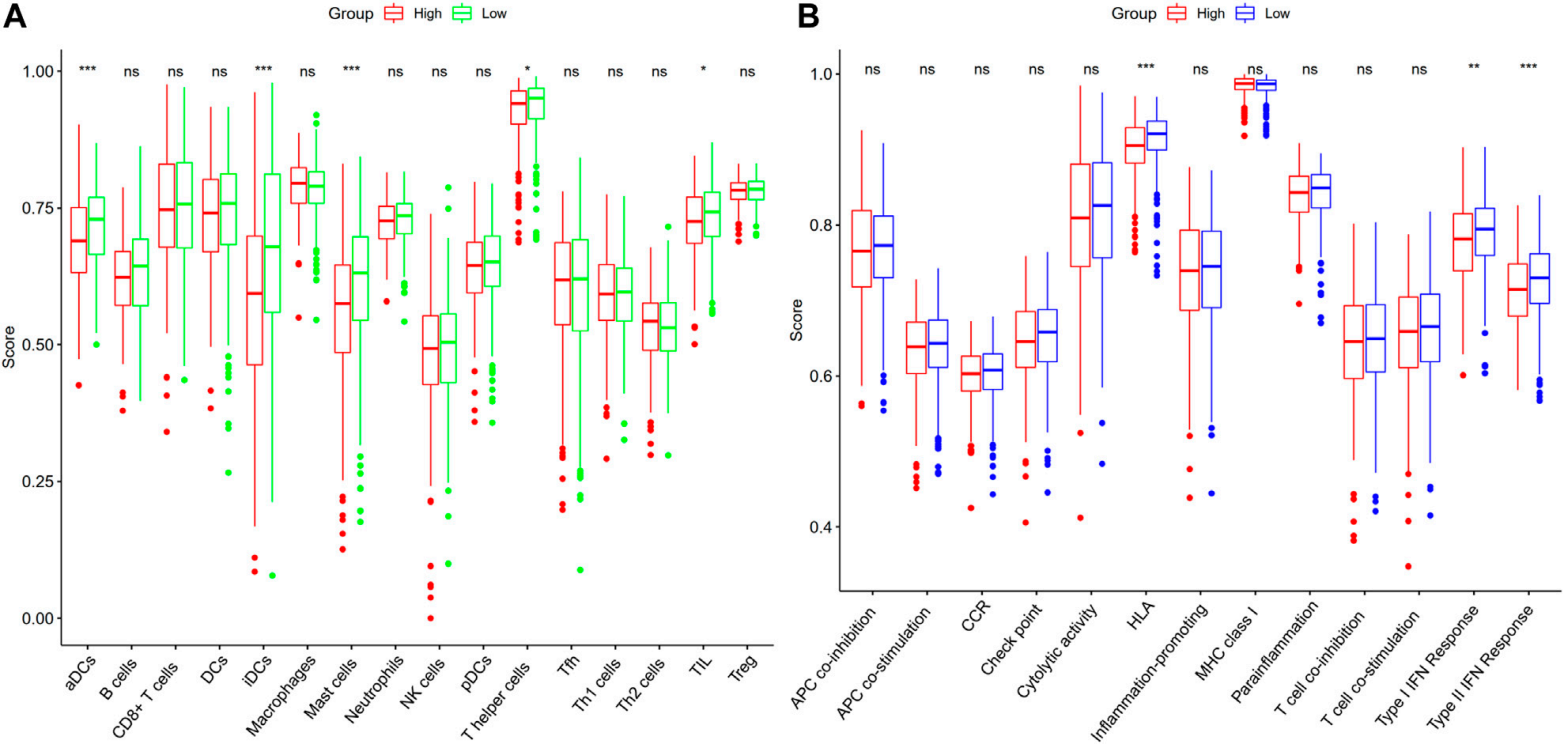
ssGSEA score comparison for immune cells and immune pathways. **(A)** Comparison of the enrichment values of 16 different kinds of immune cells in the TCGA dataset between patients at high (red box) and low risks (green box). **(B)** Patients in the high-risk (red box) and low-risk (blue box) groups in the TCGA cohort were compared on the basis of the enrichment values of 13 distinct immune-associated pathways. iDCs, immature dendritic cells; aDCs, activated dendritic cells; TIL, tumor-infiltrating lymphocyte; DCs, dendritic cells; IFN, interferon; NK cells, natural killer cells; MHC, major histocompatibility complex; Th1 cells, type 1 T helper cells; Tfh, T follicular helper cell; Th2 cells, type 2 T helper cells; APC, antigen processing cell; Treg, regulatory T cell; HLA, human leukocyte antigen; CCR, cytokine-cytokine receptor; pDCs, plasmacytoid dendritic cells; ns, not significant; *p < 0.05; **p < 0.01; ***p < 0.001.

With the help of the ssGSEA, we were able to additionally evaluate the enrichment scores for 16 distinct kinds of immune cells and the functioning of 13 immune-associated pathways between the high- and low-risk subgroups in the TCGA dataset. In the TCGA cohort, we ascertained that the high-risk subgroup exhibited reduced infiltrating levels of immune cells including T helper cells, immature dendritic cells (iDCs), mast cells, activated dendritic cells (aDCs), and tumor-infiltrating lymphocytes (TILs) in contrast with the low-risk subgroup (Figure 6A). Within the TCGA cohort, three immunological pathways were shown to be less active in the high-risk subgroup as opposed to the low-risk subgroup, including the human leukocyte antigen (HLA), type I interferon (IFN) response, and type II IFN response (Figure 6B).

## Discussion

In this work, we initially evaluated the mRNA levels of 13 presently recognized cuproptosis-associated genes in LUAD and normal specimens and discovered a differential expression among several of them. There were interactions and correlations among these genes. Nonetheless, the three clusters generated from the consensus clustering assessment premised on the cuproptosis-related genes, on the other hand, did not exhibit any evident significant differences in clinical parameters or OS time. We conducted univariate and LASSO Cox regression analyses on these cuproptosis-associated genes in order to test their prognostic significance further. We then created a 6-gene risk signature using the results of this investigation. After that, a risk score was established, and the patients were categorized into two subgroups: low-risk and high-risk subgroups. Cox regression analyses, both univariate and multivariate, were employed to establish if the risk score resulting from the gene signature model independently served as a prognostic indicator. In accordance with the functional analyses, the DEGs that distinguished the low-risk patients from the high-risk ones were linked to cilium-related function. The infiltration of immune cells and the activation of signaling pathways in the low- and high-risk subgroups were also examined. We discovered reduced infiltrating levels of immune cells in the high-risk subgroup, including aDCs, iDCs, T helper cells, mast cells, and TILs, as opposed to the low-risk subgroup. HLA, type I IFN response and type II IFN response showed lower activity in the high-risk in contrast with the low-risk subgroup.

In recent years, PCD was identified as having a dual role in the genesis of tumors and therapeutic processes. As a result of the abundant supply of inflammatory mediators generated by PCD, normal cells are activated, ultimately resulting in their transition into cancer cells. The enhancement of tumor cell PCD, on the other hand, may provide a novel treatment target (Lu et al., 2017; Karki and Kanneganti, 2019; Xia et al., 2019; Al et al., 2021; Koren and Fuchs, 2021). Cell death performs an instrumental function in the origin and treatment of cancer, while several studies have established tumor prognostic models associated with cell death (Cai et al., 2021; Fu et al., 2021; Shao et al., 2021; Zhao et al., 2021).

Cuproptosis, copper-dependent cell death presented by a recent article, as a novel form of PCD (Tsvetkov et al., 2022). Copper is a double-edged sword as it is required as an enzyme cofactor, but it may also be poisonous at even modest intracellular levels, leading to cell death (Ge et al., 2022). As a consequence of cuproptosis, which targets lipoylated TCA cycle proteins, lipoylated protein aggregation and consequent iron-sulfur cluster protein depletion occurs, resulting in proteotoxic stress and eventually inducing the death of cells. These new results may also invigorate studies exploring the use of copper to treat cancer (Kahlson and Dixon, 2022; Tsvetkov et al., 2022). At present, the relevant mechanism research of cuproptosis and tumors should be further deepened. Nonetheless, it may be necessary to first investigate how cuproptosis-associated genes interact with one another and if they are linked to the survival time of patients with LUAD.

Our research established a signature consisting of six cuproptosis-related genes (DBT, DLAT, DLD, DLST, LIPT1, and PDHA1) and discovered that it might anticipate OS among LUAD patients using this signature. Mutation of zebrafish DBT could result in motor dysfunction (Friedrich et al., 2012). DLAT is related to liver cancer metabolism and autophagy with chemotherapeutic resistance (Huang et al., 2019). In patients with head and neck cancer, DLD has been shown to modulate cystine deprivation-mediated ferroptosis (Shin et al., 2020). DLD inhibition could have resulted in lower levels of TCA cycle downstream metabolites, and downmodulation of DLD promoted autophagy in melanoma cells, as well as inhibiting tumor growth and proliferation *in vivo* (Yumnam et al., 2021). The metabolic heterogeneity in TCA cycle utilization amongst triple-negative breast cancer patients is dictated by DLST reliance (Shen et al., 2021). Germline DLST variants promote epigenetic modifications in pheochromocytoma-paraganglioma (Buffet et al., 2021). LIPT1 plays an important role in metabolic regulation (Stowe et al., 2018; Ni et al., 2019). PDHA1 gene deletion in prostate cancer cells causes metabolic remodeling, with the cells becoming more glutamine-reliant (Li et al., 2016). When PDHA1 is downmodulated in breast cancer, the oncoprotein hepatitis B X-interacting protein may help to drive glucose metabolic remodeling (Liu et al., 2015). These genes have some association with the origin and treatment of cancer. However, these genes in the prognostic model, that are cuproptosis promoters or executors should be further studied. Although not all of these promoters and executors were linked to an improved prognosis among patients with LUAD in our analysis, none was linked to a dismal prognosis. It is yet uncertain how these genes interact with one another during cuproptosis, and additional research is warranted.

Cuproptosis has not been thoroughly investigated up to today. A variety of PCD strategies might coexist and interface with one another as tumors grow and progress (Fritsch et al., 2019). For instance, among the 6 cuproptosis-related genes, DLAT is associated with autophagy (Xu et al., 2020); DLD is also known as key regulator ferroptosis (Lu et al., 2017; Park et al., 2018); PDHA1 is closely related to apoptosis (Kwak et al., 2020; Jin et al., 2021). It has been shown that the PCD-related gene correlated with the prognosis of tumor patients, whose mechanism is related to immune cell infiltration (Hong et al., 2021; Ye et al., 2021). Thus, cuproptosis-related genes are certainly associated with other PCD, with the possibility that some genes are involved in multiple ways of PCD. Furthermore, it is possible that low levels of antitumor immunity are responsible for the unfavorable survival results in high-risk patients. They may have an intact cell plasma membrane with no release of contents, which would induce indirect inflammatory reactions, or they could have the opposite properties (Galluzzi et al., 2018; Khan et al., 2021). So, we also performed the immune infiltration enrichment analyses. We found that the infiltration levels of aDCs, iDCs, mast cells, T helper cells and TILs, type I IFN, and type II IFN responses showed lower activity in the high-risk group as opposed to the low-risk group, indicating that the high-risk patients may experience immune system dysfunction. However, no differences were identified between the levels of other main anti-tumor infiltrating immune cells between the two groups. Unlike other PCD, immune infiltration might perform a smaller function in cuproptosis-related genes correlated with the prognosis of LUAD patients, and other deep mechanisms may be involved.

We assessed the DEGs between distinct risk groups and discovered that the DEGs were predominantly implicated in cilium related function, such as movement based on microtubules, cilium movement, flagellum or cilium-dependent cell motility, cell motility that is mediated by the cilium, cell motility mediated by cilium movement, motile cilium, 9 + 2 motile cilium, and so on. Outside the cell surface, the main cilium is an antenna-like structure that extends beyond the cell membrane. Cilium performs a crucial function in the modulation of cell-signaling transduction, which has an impact on the capacity of cells to proliferate, differentiate, and migrate. Ciliary impairments result in ciliopathies, and ciliary dysregulation performs a critical function in the genesis and progression of cancer. Some cancer cells may undergo growth suppression by restoring the cilia (Wang et al., 2021). Oncogenic signaling pathways, as well as certain specific anticancer treatments, may either stimulate or suppress ciliation. Interactions between the genomic profiles of tumor cells, medication therapy, and ciliary signaling in the tumor microenvironment are expected to have an impact on tumor progression and responsiveness to treatment (Liu et al., 2018). Ciliary disintegration abnormalities are generally linked to the genesis of tumors. The identification of modulators of ciliary disassembly and mitosis is critical in the search for targeted therapies for cancers that are related to these modulators (Doornbos and Roepman, 2021). Ciliogenesis and Hedgehog signaling are suppressed downstream of KRAS all through acinar-ductal metaplasia in mice, which might be employed as a method to limit the progression of early lesions and, therefore, the advancement to pancreatic ductal adenocarcinoma (Bangs et al., 2020). At present, there are still few studies on cilium and tumors, and cilium has the potential to participate in the development of tumors together with cuproptosis, thus affecting the prognosis. The specific mechanisms deserve further investigation.

Cuproptosis is a topic that has received little recent attention, particularly in terms of its mechanisms in LUAD. Our research developed a gene signature associated with cuproptosis. Using these cuproptosis-related genes, we were able to conduct a preliminary analysis of their predictive significance and establish a theoretical foundation for further investigation. We established *p* < 0.3 as the cut-off value to avoid missing important genes and identified 6 genes associated with survival for subsequent evaluation. Therefore, further accurate verification and large sample size verification should be studied in the future. The absence of data, however, prevented us from concluding if these genes also perform similar roles in various cuproptosis pathways in LUAD, and this is an issue that warrants additional investigation.

## Conclusion

In summary, our research indicated that cuproptosis is strongly linked to LUAD since the expression levels of most cuproptosis-associated genes differed between normal and LUAD specimens. Furthermore, the score derived from our risk signature, which was on the basis of six cuproptosis-associated genes, was shown to independently serve as a risk indicated for anticipating LUAD outcomes in the TCGA cohort. The DEGs that distinguished the low-risk patients from the high-risk ones were linked to the cilium. LUAD patients’ prognoses may now be predicted using a unique gene signature identified in this work. This discovery also offers a substantial foundation for future research into the links between cuproptosis-associated genes and cilium in LUAD patients.

## Data Availability

The data could be download from UCSC Xena (http://xena.ucsc.edu), some data generated or analyzed during this study are included in the article/Supplementary Material, further inquiries can be directed to the corresponding authors.

## References

[B1] Ahmed A. Tait S. (2020). Targeting immunogenic cell death in cancer. Mol. Oncol. 14 (12), 2994–3006. 10.1002/1878-0261.12851 33179413 PMC7718954

[B2] Al M. A. Mimi A. A. Aziz M. A. Zaeem M. Ahmed T. Munir F. (2021). Role of pyroptosis in cancer and its therapeutic regulation. Eur. J. Pharmacol. 910, 174444. 10.1016/j.ejphar.2021.174444 34453928

[B3] Ali A. Goffin J. R. Arnold A. Ellis P. M. (2013). Survival of patients with non-small-cell lung cancer after a diagnosis of brain metastases. Curr. Oncol. 20 (4), e300. 10.3747/co.20.1481 23904768 PMC3728058

[B4] Bangs F. K. Miller P. O'Neill E. (2020). Ciliogenesis and Hedgehog signalling are suppressed downstream of KRAS during acinar-ductal metaplasia in mouse. Dis. Model. Mech. 13 (7), dmm044289. 10.1242/dmm.044289 32571902 PMC7406310

[B5] Barta J. A. Powell C. A. Wisnivesky J. P. (2019). Global epidemiology of lung cancer. Ann. Glob. Health 85 (1), 8. 10.5334/aogh.2419 30741509 PMC6724220

[B6] Buffet A. Zhang J. Rebel H. Corssmit E. Jansen J. C. Hensen E. F. (2021). Germline DLST variants promote epigenetic modifications in pheochromocytoma-paraganglioma. J. Clin. Endocrinol. Metab. 106 (2), 459–471. 10.1210/clinem/dgaa819 33180916

[B7] Cai H. J. Zhuang Z. C. Wu Y. Zhang Y. Y. Liu X. Zhuang J. F. (2021). Development and validation of a ferroptosis-related lncRNAs prognosis signature in colon cancer. Bosn. J. Basic Med. Sci. 21 (5), 569–576. 10.17305/bjbms.2020.5617 33714257 PMC8381210

[B8] Chen C. Chen S. Hu X. Wang J. Wen T. Fu J. (2020). Effects of autophagy-associated genes on the prognosis for lung adenocarcinoma. Transl. Cancer Res. 9 (3), 1947–1959. 10.21037/tcr.2020.02.07 35117541 PMC8798140

[B9] Chin L. Hahn W. C. Getz G. Meyerson M. (2011). Making sense of cancer genomic data. Genes Dev. 25 (6), 534–555. 10.1101/gad.2017311 21406553 PMC3059829

[B10] Doornbos C. Roepman R. (2021). Moonlighting of mitotic regulators in cilium disassembly. Cell. Mol. Life Sci. 78 (11), 4955–4972. 10.1007/s00018-021-03827-5 33860332 PMC8233288

[B11] Engebretsen S. Bohlin J. (2019). Statistical predictions with glmnet. Clin. Epigenetics. 11 (1), 123. 10.1186/s13148-019-0730-1 31443682 PMC6708235

[B12] Friedrich T. Lambert A. M. Masino M. A. Downes G. B. (2012). Mutation of zebrafish dihydrolipoamide branched-chain transacylase E2 results in motor dysfunction and models maple syrup urine disease. Dis. Model. Mech. 5 (2), 248–258. 10.1242/dmm.008383 22046030 PMC3291646

[B13] Fritsch M. Gunther S. D. Schwarzer R. Albert M. C. Schorn F. Werthenbach J. P. (2019). Caspase-8 is the molecular switch for apoptosis, necroptosis and pyroptosis. Nature 575 (7784), 683–687. 10.1038/s41586-019-1770-6 31748744

[B14] Fu D. Zhang B. Wu S. Zhang Y. Xie J. Ning W. (2021). Prognosis and characterization of immune microenvironment in acute myeloid leukemia through identification of an autophagy-related signature. Front. Immunol. 12, 695865. 10.3389/fimmu.2021.695865 34135913 PMC8200670

[B15] Galluzzi L. Vitale I. Aaronson S. A. Abrams J. M. Adam D. Agostinis P. (2018). Molecular mechanisms of cell death: recommendations of the nomenclature committee on cell death 2018. Cell Death Differ. 25 (3), 486–541. 10.1038/s41418-017-0012-4 29362479 PMC5864239

[B16] Garg A. D. Agostinis P. (2017). Cell death and immunity in cancer: from danger signals to mimicry of pathogen defense responses. Immunol. Rev. 280 (1), 126–148. 10.1111/imr.12574 29027218

[B17] Ge E. J. Bush A. I. Casini A. Cobine P. A. Cross J. R. Denicola G. M. (2022). Connecting copper and cancer: from transition metal signalling to metalloplasia. Nat. Rev. Cancer. 22 (2), 102–113. 10.1038/s41568-021-00417-2 34764459 PMC8810673

[B18] Goldman M. J. Craft B. Hastie M. Repecka K. Mcdade F. Kamath A. (2020). Visualizing and interpreting cancer genomics data via the Xena platform. Nat. Biotechnol. 38 (6), 675–678. 10.1038/s41587-020-0546-8 32444850 PMC7386072

[B19] Grossman R. L. Heath A. P. Ferretti V. Varmus H. E. Lowy D. R. Kibbe W. A. (2016). Toward a shared vision for cancer genomic data. N. Engl. J. Med. 375 (12), 1109–1112. 10.1056/NEJMp1607591 27653561 PMC6309165

[B20] Hanzelmann S. Castelo R. Guinney J. (2013). Gsva: gene set variation analysis for microarray and RNA-seq data. BMC Bioinforma. 14, 7. 10.1186/1471-2105-14-7 PMC3618321 23323831

[B21] Hong Y. Lin M. Ou D. Huang Z. Shen P. (2021). A novel ferroptosis-related 12-gene signature predicts clinical prognosis and reveals immune relevancy in clear cell renal cell carcinoma. BMC Cancer 21 (1), 831. 10.1186/s12885-021-08559-0 34281531 PMC8290606

[B22] Huang X. Gan G. Wang X. Xu T. Xie W. (2019). The HGF-MET axis coordinates liver cancer metabolism and autophagy for chemotherapeutic resistance. Autophagy 15 (7), 1258–1279. 10.1080/15548627.2019.1580105 30786811 PMC6613896

[B23] Jin L. Cho M. Kim B. S. Han J. H. Park S. Lee I. K. (2021). Drug evaluation based on phosphomimetic PDHA1 reveals the complexity of activity-related cell death in A549 non-small cell lung cancer cells. BMB Rep. 54 (11), 563–568. 10.5483/bmbrep.2021.54.11.101 34488935 PMC8633525

[B24] Kahlson M. A. Dixon S. J. (2022). Copper-induced cell death. Science 375 (6586), 1231–1232. 10.1126/science.abo3959 35298241

[B25] Karki R. Kanneganti T. D. (2019). Diverging inflammasome signals in tumorigenesis and potential targeting. Nat. Rev. Cancer. 19 (4), 197–214. 10.1038/s41568-019-0123-y 30842595 PMC6953422

[B26] Khan I. Yousif A. Chesnokov M. Hong L. Chefetz I. (2021). A decade of cell death studies: breathing new life into necroptosis. Pharmacol. Ther. 220, 107717. 10.1016/j.pharmthera.2020.107717 33164841

[B27] Koren E. Fuchs Y. (2021). Modes of regulated cell death in cancer. Cancer Discov. 11 (2), 245–265. 10.1158/2159-8290.CD-20-0789 33462123

[B28] Kwak C. H. Jin L. Han J. H. Han C. W. Kim E. Cho M. (2020). Ilimaquinone induces the apoptotic cell death of cancer cells by reducing pyruvate dehydrogenase kinase 1 activity. Int. J. Mol. Sci. 21 (17), E6021. 10.3390/ijms21176021 32825675 PMC7504051

[B29] Li Y. Li X. Li X. Zhong Y. Ji Y. Yu D. (2016). PDHA1 gene knockout in prostate cancer cells results in metabolic reprogramming towards greater glutamine dependence. Oncotarget 7 (33), 53837–53852. 10.18632/oncotarget.10782 27462778 PMC5288225

[B30] Lin W. Chen Y. Wu B. Chen Y. Li Z. (2021). Identification of the pyroptosisrelated prognostic gene signature and the associated regulation axis in lung adenocarcinoma. Cell Death Discov. 7 (1), 161. 10.1038/s41420-021-00557-2 34226539 PMC8257680

[B31] Liu F. Zhang W. You X. Liu Y. Li Y. Wang Z. (2015). The oncoprotein HBXIP promotes glucose metabolism reprogramming via downregulating SCO2 and PDHA1 in breast cancer. Oncotarget 6 (29), 27199–27213. 10.18632/oncotarget.4508 26309161 PMC4694983

[B32] Liu H. Kiseleva A. A. Golemis E. A. (2018). Ciliary signalling in cancer. Nat. Rev. Cancer. 18 (8), 511–524. 10.1038/s41568-018-0023-6 29802351 PMC6448793

[B33] Lu B. Chen X. B. Ying M. D. He Q. J. Cao J. Yang B. (2017). The role of ferroptosis in cancer development and treatment response. Front. Pharmacol. 8, 992. 10.3389/fphar.2017.00992 29375387 PMC5770584

[B34] Lu Y. Luo X. Wang Q. Chen J. Zhang X. Li Y. (2022). A novel necroptosis-related lncRNA signature predicts the prognosis of lung adenocarcinoma. Front. Genet. 13, 862741. 10.3389/fgene.2022.862741 35368663 PMC8969905

[B35] Moujalled D. Strasser A. Liddell J. R. (2021). Molecular mechanisms of cell death in neurological diseases. Cell Death Differ. 28 (7), 2029–2044. 10.1038/s41418-021-00814-y 34099897 PMC8257776

[B36] Ni M. Solmonson A. Pan C. Yang C. Li D. Notzon A. (2019). Functional assessment of lipoyltransferase-1 deficiency in cells, mice, and humans. Cell Rep. 27 (5), 13761386.e6. 10.1016/j.celrep.2019.04.005 PMC7351313 31042466

[B37] Park S. Oh J. Kim M. Jin E. J. (2018). Bromelain effectively suppresses Kras-mutant colorectal cancer by stimulating ferroptosis. Anim. Cells Syst. 22 (5), 334–340. 10.1080/19768354.2018.1512521 PMC6171431 30460115

[B38] Ritchie M. E. Phipson B. Wu D. Hu Y. Law C. W. Shi W. (2015). Limma powers differential expression analyses for RNA-sequencing and microarray studies. Nucleic Acids Res. 43 (7), e47. 10.1093/nar/gkv007 25605792 PMC4402510

[B39] Shao W. Yang Z. Fu Y. Zheng L. Liu F. Chai L. (2021). The pyroptosis-related signature predicts prognosis and indicates immune microenvironment infiltration in gastric cancer. Front. Cell Dev. Biol. 9, 676485. 10.3389/fcell.2021.676485 34179006 PMC8226259

[B40] Shao X. Wang X. Guo X. Jiang K. Ye T. Chen J. (2019). STAT3 contributes to oncolytic newcastle disease virus-induced immunogenic cell death in melanoma cells. Front. Oncol. 9, 436. 10.3389/fonc.2019.00436 31192135 PMC6548873

[B41] Shen N. Korm S. Karantanos T. Li D. Zhang X. Ritou E. (2021). DLST-dependence dictates metabolic heterogeneity in TCA-cycle usage among triple-negative breast cancer. Commun. Biol. 4 (1), 1289. 10.1038/s42003-021-02805-8 34785772 PMC8595664

[B42] Shin D. Lee J. You J. H. Kim D. Roh J. L. (2020). Dihydrolipoamide dehydrogenase regulates cystine deprivation-induced ferroptosis in head and neck cancer. Redox Biol. 30, 101418. 10.1016/j.redox.2019.101418 31931284 PMC6957841

[B43] Stowe R. C. Sun Q. Elsea S. H. Scaglia F. (2018). LIPT1 deficiency presenting as early infantile epileptic encephalopathy, Leigh disease, and secondary pyruvate dehydrogenase complex deficiency. Am. J. Med. Genet. A 176 (5), 1184–1189. 10.1002/ajmg.a.38654 29681092

[B44] Strasser A. Vaux D. L. (2020). Cell death in the origin and treatment of cancer. Mol. Cell. 78 (6), 1045–1054. 10.1016/j.molcel.2020.05.014 32516599

[B45] Szklarczyk D. Gable A. L. Nastou K. C. Lyon D. Kirsch R. Pyysalo S. (2021). The STRING database in 2021: customizable protein-protein networks, and functional characterization of user-uploaded gene/measurement sets. Nucleic Acids Res. 49 (D1), D605–D612. 10.1093/nar/gkaa1074 33237311 PMC7779004

[B46] Tang R. Xu J. Zhang B. Liu J. Liang C. Hua J. (2020). Ferroptosis, necroptosis, and pyroptosis in anticancer immunity. J. Hematol. Oncol. 13 (1), 110. 10.1186/s13045-020-00946-7 32778143 PMC7418434

[B47] Tibshirani R. (1997). The lasso method for variable selection in the Cox model. Stat. Med. 16 (4), 385–395. 10.1002/(sici)1097-0258(19970228)16:4<385::aid-sim380>3.0.co;2-3 9044528

[B48] Tsvetkov P. Coy S. Petrova B. Dreishpoon M. Verma A. Abdusamad M. (2022). Copper induces cell death by targeting lipoylated TCA cycle proteins. Science 375 (6586), 1254–1261. 10.1126/science.abf0529 35298263 PMC9273333

[B49] Walter W. Sanchez-Cabo F. Ricote M. (2015). GOplot: an R package for visually combining expression data with functional analysis. Bioinformatics 31 (17), 2912–2914. 10.1093/bioinformatics/btv300 25964631

[B50] Wang B. Liang Z. Liu P. (2021). Functional aspects of primary cilium in signaling, assembly and microenvironment in cancer. J. Cell. Physiol. 236 (5), 3207–3219. 10.1002/jcp.30117 33107052 PMC7984063

[B51] Wilkerson M. D. Hayes D. N. (2010). ConsensusClusterPlus: a class discovery tool with confidence assessments and item tracking. Bioinformatics 26 (12), 1572–1573. 10.1093/bioinformatics/btq170 20427518 PMC2881355

[B52] Wu T. Hu E. Xu S. Chen M. Guo P. Dai Z. (2021). clusterProfiler 4.0: a universal enrichment tool for interpreting omics data. Innovation. 2 (3), 100141. 10.1016/j.xinn.2021.100141 34557778 PMC8454663

[B53] Xia X. Wang X. Cheng Z. Qin W. Lei L. Jiang J. (2019). The role of pyroptosis in cancer: pro-cancer or pro-"host. Cell Death Dis. 10 (9), 650. 10.1038/s41419-019-1883-8 31501419 PMC6733901

[B54] Xu Y. Shen J. Ran Z. (2020). Emerging views of mitophagy in immunity and autoimmune diseases. Autophagy 16 (1), 3–17. 10.1080/15548627.2019.1603547 30951392 PMC6984455

[B55] Yao J. Chen X. Liu X. Li R. Zhou X. Qu Y. (2021). Characterization of a ferroptosis and iron-metabolism related lncRNA signature in lung adenocarcinoma. Cancer Cell Int. 21 (1), 340. 10.1186/s12935-021-02027-2 34217273 PMC8254945

[B56] Ye Y. Dai Q. Qi H. (2021). A novel defined pyroptosis-related gene signature for predicting the prognosis of ovarian cancer. Cell Death Discov. 7 (1), 71. 10.1038/s41420-021-00451-x 33828074 PMC8026591

[B57] Yu G. Wang L. G. Han Y. He Q. Y. (2012). ClusterProfiler: an R package for comparing biological themes among gene clusters. OMICS 16 (5), 284–287. 10.1089/omi.2011.0118 22455463 PMC3339379

[B58] Yumnam S. Kang M. C. Oh S. H. Kwon H. C. Kim J. C. Jung E. S. (2021). Downregulation of dihydrolipoyl dehydrogenase by UVA suppresses melanoma progression via triggering oxidative stress and altering energy metabolism. Free Radic. Biol. Med. 162, 77–87. 10.1016/j.freeradbiomed.2020.11.037 33279616

[B59] Zhang X. Lou Y. Zheng X. Wang H. Sun J. Dong Q. (2015). Wnt blockers inhibit the proliferation of lung cancer stem cells. Drug Des. devel. Ther. 9, 2399–2407. 10.2147/DDDT.S76602 PMC4423515 25960639

[B60] Zhao Z. Liu H. Zhou X. Fang D. Ou X. Ye J. (2021). Necroptosis-related lncRNAs: predicting prognosis and the distinction between the cold and hot tumors in gastric cancer. J. Oncol. 2021, 6718443. 10.1155/2021/6718443 34790235 PMC8592775

